# Coproducing a Cochrane Qualitative Evidence Synthesis: Process, Outcomes, and Reflections on Power

**DOI:** 10.1002/cesm.70025

**Published:** 2025-03-18

**Authors:** Bronwen Merner, Rebecca Ryan

**Affiliations:** ^1^ Department of Public Health Cochrane Consumers and Communication, Centre for Health Communication and Participation, La Trobe University Melbourne Victoria Australia

## Abstract

Reflecting a broader movement toward knowledge democratization, coproducing Cochrane evidence with interest holders outside universities is increasingly encouraged. However, only limited research exists on the approaches used to coproduce Cochrane reviews. Furthermore, the outcomes of coproduction are rarely described. In this commentary, we aim to address these gaps by describing the process and outcomes of coproduction used in a recently published Cochrane qualitative evidence synthesis (QES). We also reflect on power imbalances in our coproduction approach and how these could be minimized in future review coproduction activities.

## 1 Background to the Review

In 2023, we were members of a team, led by B. M., that published a coproduced Cochrane qualitative evidence synthesis (QES) entitled “Consumers' and health providers' views and perceptions of partnering to improve health services design, delivery and evaluation” [1].

The review originated from the results of a formal priority‐setting process undertaken by Cochrane Consumers and Communication (CCC), the Cochrane editorial group responsible for publishing reviews about health communication and consumer involvement [2]. The priority‐setting process identified the improvement of person‐centered care as a priority area for future Cochrane reviews. When we volunteered to undertake a review on this topic, CCC encouraged us (and authors of other priority reviews) to involve consumers and other interest holders in producing the review to enhance the relevance of the results to policy and practice [3]. Though we were both experienced researchers, this was our first experience of research coproduction.

## 2 Coproduction Approach

### 2.1 Recruitment of Panel Members

As coproduction lead, B. M. selected an approach informed by Pollock and colleagues, who coproduced a Cochrane intervention review update [4]. Pollock's approach involved recruiting an interest holder panel to contribute to review planning and interpreting the results. Subsequently, 18 interest holder panel members were recruited for our review, using a combination of closed (e.g., professional networks) and open (advertising through a person‐centered care network) recruitment techniques [5]. The panel was limited to Australia only so more flexibility in online meeting times could be offered (e.g., after working hours) and also to maintain the possibility of a face‐to‐face meeting. The final panel consisted of six consumers, six health service representatives, and six policymakers from across the country.

Once established, the research team partnered with the panel, sharing power and responsibility, from the start of the review (topic selection) to the end (publication). Table 1 provides an overview of our coproduction approach, showing we met with the panel at four key stages in the review process: (1) topic selection, (2) protocol development, (3) screening, and (4) analysis and implications.

**Table 1:** Summary of Coproduction process used in Merner et al. [1].

Review stage	Involvement aim	Description of involvement	Level of involvement in decision‐making (IAP2)	Involvement outcomes
Topic selection (Meeting 1)	Choose type of review Seek topic suggestions	Within the broad priority area of improving person‐centered care, we aimed to develop a specific review question. During an online group meeting, we discussed how different review types addressed different review questions (e.g., intervention reviews are best for questions about “what to do” whereas QES addresses questions about “how to do” something). We sought interest holders' ideas on potential topics within the person‐centered care area that the review could explore. Following the meeting, interest holders completed a survey in which they ranked their preferred review type and selected their preferred topics from a short list provided to them.	Empower—panel's preferred review type (QES) was implemented	Immediate outcome: Change in review scope
				QES decided as review type Topic suggestions provided
				Intermediate outcome: Creating key relationships
Protocol development (Meeting 2)	Improve the relevance of the research question, selection criteria, and search terms	Following the scoping of review topics from the first meeting, researchers determined that consumer engagement in health service governance would be the most fruitful area for a Cochrane Review. Researchers developed a draft protocol on this topic and sent this (as well as the agenda and discussion questions) to interest holders 1 week prior to the meeting. During the meeting, each interest holder was asked for feedback about the research question, selection criteria, and search terms.	Collaborate—advice was incorporated into protocol methods to the maximum extent possible	Immediate outcome: Change in review methods
				Objective added: Development of best practice principles Refined selection criteria Restructured Background to improve clarity New terms added to search strategy
				Intermediate outcome: Sustaining key relationships
Review—screening (Meeting 3)	Build capacity of stakeholders to participate in review tasks Improve the relevance of the screening process	In a face‐to‐face meeting, a presentation was given to the interest holders about review processes and how to screen an article against selection criteria. Interest holders screened full‐text articles in small groups.	Collaborate—screening decisions on individual papers were made by consensus between researchers and interest holders.	Immediate outcome: Change in review methods
				Refined application of selection criteria Articles screened by researchers and interest holders
				Intermediate outcome: Sustaining key relationships
Review—analysis and implications (Meeting 4)	Review draft findings for relevance Develop best practice principles from findings of the review to enhance translation of review findings	Interest holders were sent a draft copy of the review findings a week prior to the meeting. At the online meeting, interest holders were asked to identify any gaps in the findings. A list of potential best practice principles derived from the findings of the review was also provided. Interest holders used the list as the basis for adding, subtracting and refining the best practice principles.	Collaborate—advice was sought from panel and incorporated into interpretation of the review's findings. Panel's solutions on the development of best practice principles were incorporated to the maximum extent possible	Immediate outcome: Change in interpretation of review
				Identified a gap in the findings Developed the best practice principles
				Intermediate outcome: Sustaining key relationships

### 2.2 Meeting Preparation and Facilitation

Two weeks prior to each meeting, the agenda and discussion questions were provided to panel members to ensure they had time to consider their responses. In early panel meetings, the facilitator (a research team member) used a highly structured facilitation style to ensure that every panel member was given the opportunity to present their view. Following each meeting, the panel was provided with a record of how each recommendation from the meeting had been actioned to maximize accountability.

### 2.3 Level of Decision‐Making

The level of interest holder involvement in decision‐making for each meeting is shown in Table 1. The IAP2 Public Participation Spectrum, which ranges from minimal involvement (inform level) to high involvement (collaborate level and empower level), was used to classify the involvement level [6]. We used the IAP2 because it allows for flexibility in the level of involvement in specific decisions, rather than assuming that the involvement level is uniform across the whole project. Applying the spectrum, interest holders had the highest level of impact on decisions in Meeting 1 when they made the final decision about the type of review (empower level on the IAP2 spectrum). In subsequent meetings, decisions were more collaborative as the Cochrane review process required a combination of technical expertise (from the research team) and experience‐based knowledge (from interest holders). Thus, in these meetings, the interest holder panel's advice was incorporated to the maximum extent possible within the review's methodological restrictions.

### 2.4 Reciprocity

Reciprocity was encouraged in different ways throughout the codesign process. Panel members were offered the opportunity to develop research skills, such as study selection, and were coauthors on both the protocol and the review. The panel also provided networking opportunities. For the face‐to‐face meeting, interstate panel members' flights, accommodation, and transport were reimbursed and consumer members were remunerated with a daily sitting fee ($600 AUD).

## 3 Coproduction Outcomes

Table 1 shows a range of immediate and intermediate outcomes that were achieved through the involvement process. Although our coproduction approach was not formally evaluated due to financial and time constraints, we collated meeting documentation (agendas, minutes, voting materials) and other records (e.g., a Cochrane blog post, Cochrane Colloquium presentations) to provide evidence for the outcomes [7, 8, 9]. We drew from Ray and Miller's [10] framework of outcomes for planning, evaluating, and reporting interest holder engagement in research to report the outcomes. The framework was chosen because it included both immediate and intermediate outcomes, thereby enabling more in‐depth insights into the outcomes of engagement.

### 3.1 Immediate Outcomes

Immediate outcomes of coproduction on the review included changes in scope, methods, and interpretation. The major change in scope resulted from the interest holders deciding that a QES would be more relevant and useful than an intervention review. A major change in review methods resulting from coproduction was the inclusion of an additional objective, to develop best practice principles from the review findings, to facilitate translation into policy and practice. Finally, at the interpretation phase, interest holders identified a gap in the analysis that led the researchers to reanalyze data and revise one of the findings.

### 3.2 Intermediate Outcomes

The coproduction process also achieved an intermediate outcome relating to creating and sustaining partnerships with the interest holder panel. The strength of the partnerships formed during the review is demonstrated by the continued engagement of the interest holder panel over the 5‐year period required to complete the protocol and review [11]. Interest holders have also been involved in additional projects led by the research team [12].

## 4 Lessons From Our Experience

As demonstrated above, early and meaningful involvement through the coproduction process achieved several positive outcomes. The process transformed the type of review undertaken and the selection criteria. It also led to results that were translated into practical, best practice principles. However, as found in previous studies, the coproduction process could have been improved by more explicitly addressing power imbalances between the researchers and interest holders, particularly consumers [11, 13, 14]. In coproduction, these power imbalances stem from the historically privileged status of researchers as “authoritative knowers” leading to a greater value being placed on academic knowledge compared with other knowledge (such as experience‐based knowledge) [15]. We reflect on our experiences below as a way of advancing practice in addressing power imbalances in evidence synthesis coproduction.

Our coproduction approach would have benefited from critical reflexivity about how our positionality as White, middle‐class, university researchers influenced our assumptions about power sharing. Initially, we assumed that gathering consumers, health practitioners, and policymakers together in panel meetings would neutralize rather than exacerbate power differences. Subsequent informal discussions with consumers though revealed they felt nervous about being placed immediately into meetings with senior health service managers and policymakers. Although existing literature on coproducing evidence syntheses focuses on practical strategies for minimizing power imbalances [4, 11, 16] (e.g., working in small groups) we believe more structured reflection, using a tool such as a social identity map [17], might have prompted us to ask consumer members how they would feel most comfortable to engage rather than assuming that we already knew.

Similar to Bayliss et al. [18], who engaged interest holders in a QES, our engagement methods relied on our interest holders having high literacy levels (e.g., reading the protocol, abstracts for screening) [18]. In future, we would explore the possibility of using more inclusive engagement methods, such as World Cafes or photo voice, particularly for engaging in strategic review decisions (e.g., choice of topic or outcomes) [19, 20]. We have reflected that using engagement methods that are not equitable risks reinforcing power hierarchies that authentic coproduction seeks to dismantle [21]. Therefore, in future, we believe it appropriate only to coproduce those review tasks where interest holders could be engaged equitably. This may not be possible for technical tasks such as study selection or data extraction.

Remuneration of consumers is an important means to address power imbalances in coproduction [1]. Although we remunerated consumers for the face‐to‐face meeting, we did not have funding to pay consumers for the duration of the coproduction process. Inadequate funding is a known barrier to coproduction [22, 23]. Moving forward, we would ensure adequate funding to remunerate consumers for the whole project before commencing the review. To help with this, practical guidance is now available about budgeting for consumer involvement and some universities also advise on recommended consumer remuneration rates [24].

Finally, as others have found, more attention to practical skills, such as group facilitation and relationship‐building, may also have helped to minimize power imbalances [11]. In hindsight, undertaking our first participatory research project while simultaneously learning the rigorous Cochrane QES methods (which were also new to the research team) did not allow sufficient time to invest in practical skill development. We recommend researchers considering coproduction prioritize self‐learning or formal training in practical coproduction skills prior to beginning such research [25].

## 5 Conclusion

We have described the process of coproduction used for a Cochrane QES. The approach involved interest holders, including consumers, as panel members who contributed in different ways and at different stages of the review. An assessment of outcomes showed the coproduction process positively impacted on the immediate project and resulting review and had a sustaining effect on the partnership itself. We also reflected on the coproduction process and identified how power differentials might have been further minimized. We recommend researchers consider both the strengths and weaknesses of our approach, and the resources recommended as a means of improving their own coproduction practice in evidence syntheses.

## Author Contributions

**Bronwen Merner:** conceptualization, writing – original draft, writing – review and editing, investigation, methodology. **Rebecca Ryan:** writing – review and editing, supervision, methodology.

## Ethics Statement

The participation of the interest holder panel involved in coproducing the Cochrane QES was approved by the La Trobe University Human Research Ethics Committee (Approval Number S17‐027).

## Conflicts of Interest

The authors declare no conflicts of interest.

## Peer Review

The peer review history for this article is available at https://www.webofscience.com/api/gateway/wos/peer-review/10.1002/cesm.70025.

## Data Availability

The data that support the findings of this study are available on request from the corresponding author. The data are not publicly available due to privacy or ethical restrictions.
